# Supplementary figures and images for: DNA-based assay for calorimetric determination of protein concentrations in pure or mixed solutions
Source: PLoS One. 2024 Mar 1;19(3):e0298969. doi: 10.1371/journal.pone.0298969 (PMC10906865; doi:10.1371/journal.pone.0298969)

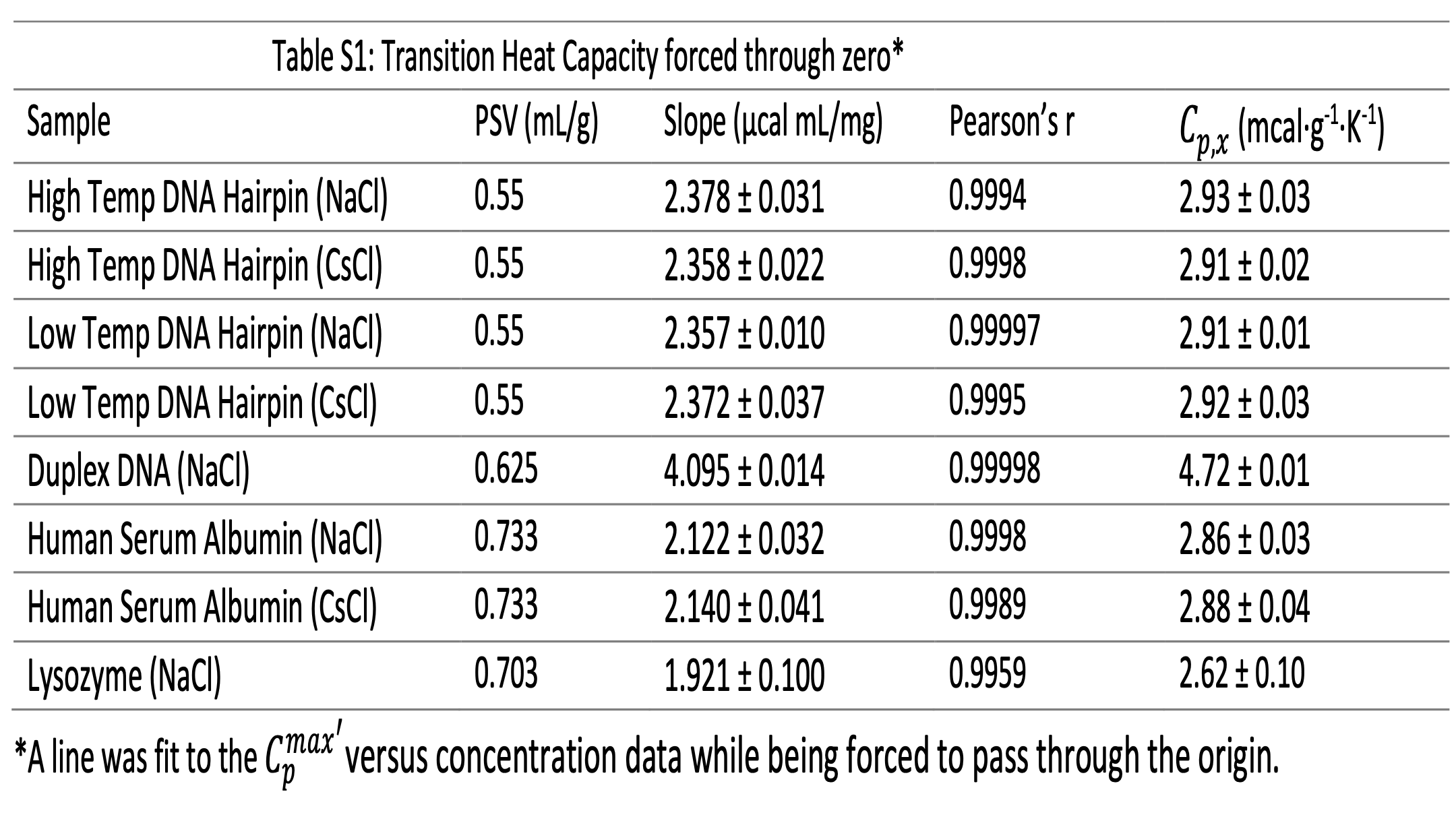

Supplement: S1 Table — (TIF) [file pone.0298969.s001.tif]

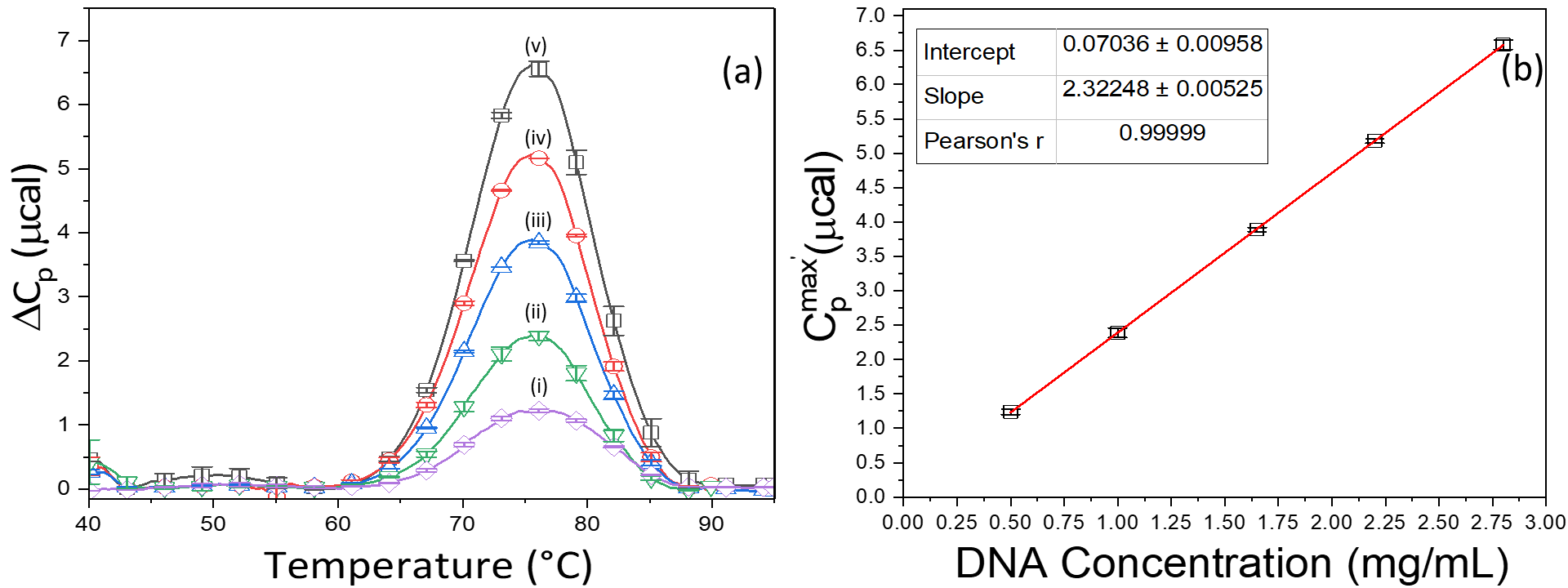

Supplement: S1 Fig — (TIF) [file pone.0298969.s002.tif]

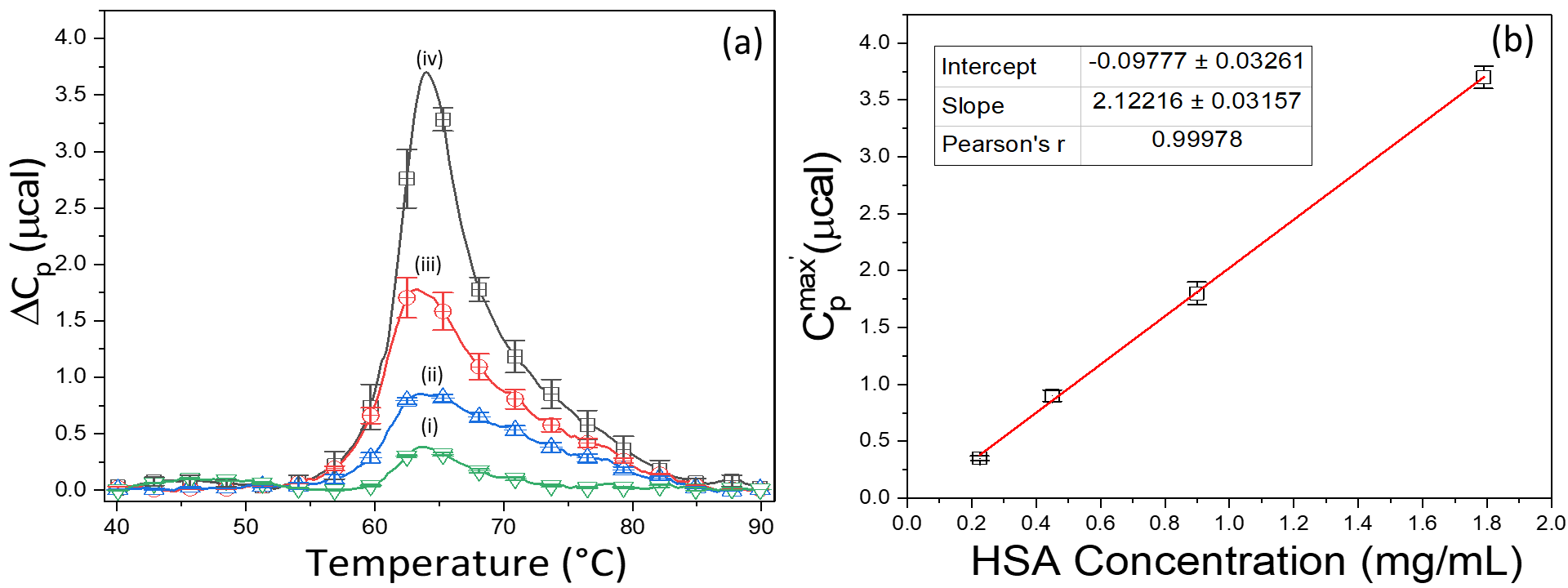

Supplement: S2 Fig — (TIF) [file pone.0298969.s003.tif]

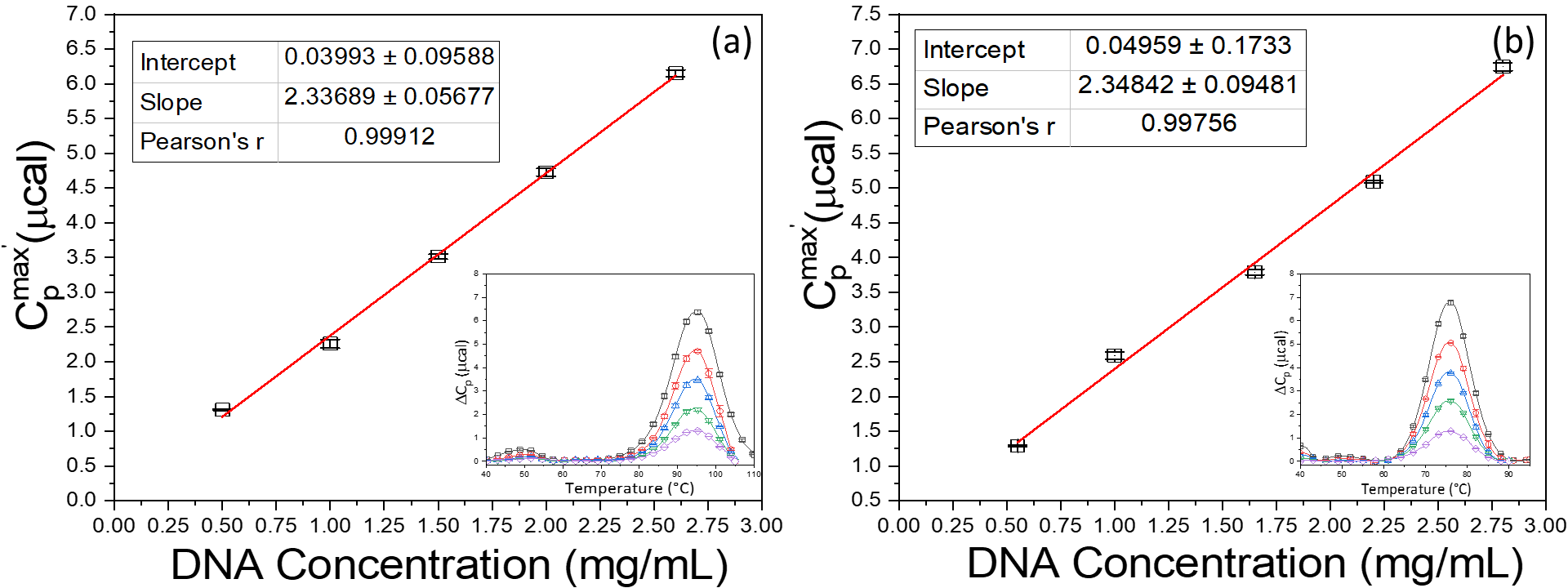

Supplement: S3 Fig — (TIF) [file pone.0298969.s004.tif]

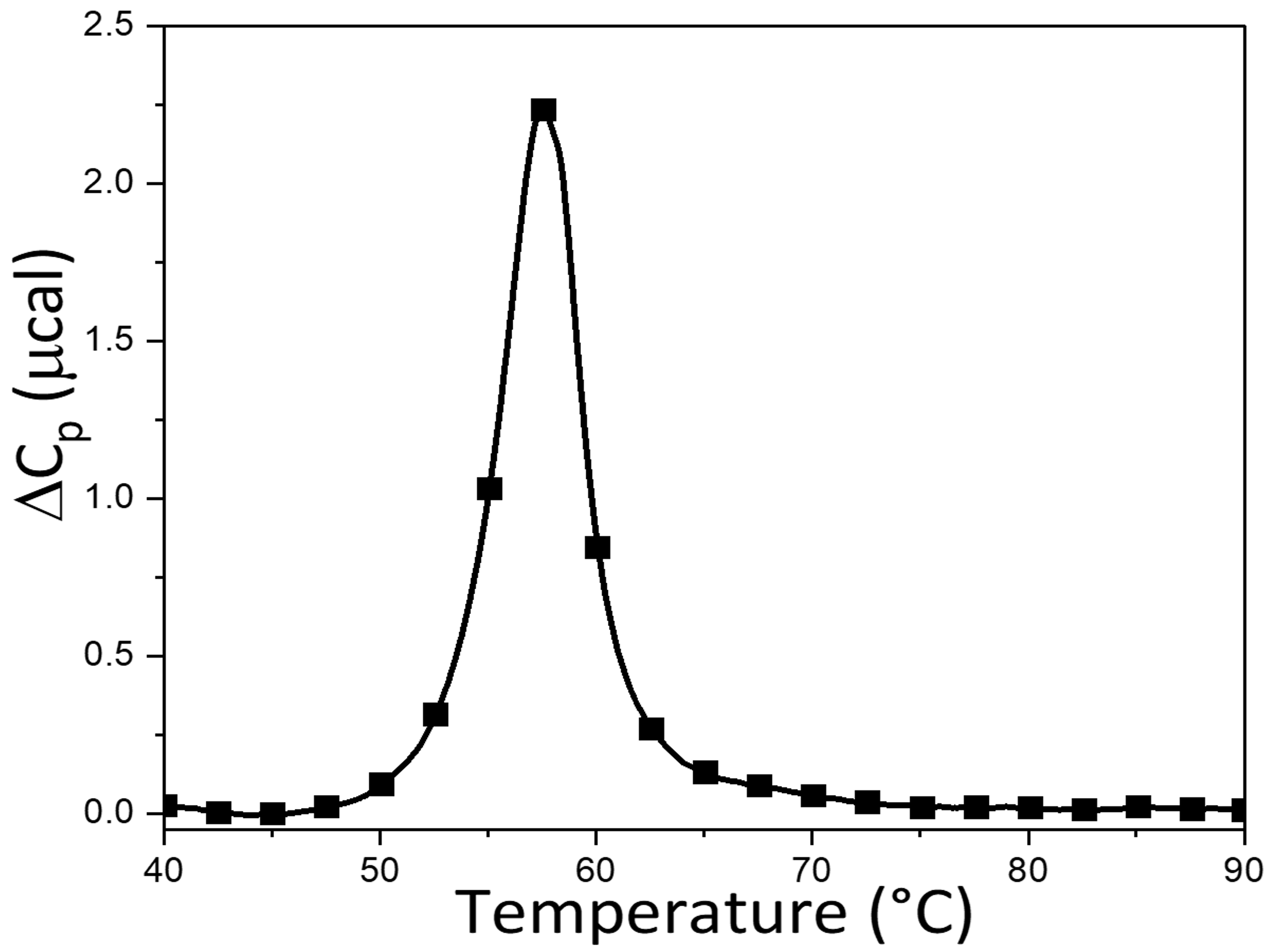

Supplement: S4 Fig — (TIF) [file pone.0298969.s005.tif]
